# Effects of fixed functional orthodontic treatment in hypodivergent and hyperdivergent class II patients—a retrospective cephalometric investigation

**DOI:** 10.1007/s00784-023-05105-z

**Published:** 2023-06-23

**Authors:** Jan Hourfar, Gero Stefan Michael Kinzinger, Linda Frye, Jörg Alexander Lisson

**Affiliations:** Department of Orthodontics, Saarland University, 66424 Homburg, Saar, Germany

**Keywords:** Hypodivergent, Hyperdivergent, Fixed functional appliance, FMA, Herbst

## Abstract

**Objective:**

To compare skeletal and dentoalveolar changes after orthodontic treatment of class II malocclusion in patients with hypodivergent and hyperdivergent growth patterns through cast splint fixed functional appliances (FFA).

**Materials and methods:**

*N* = 42 out of *n* = 47 patients with mandibular plane angles < 34° or ≥ 34° were divided into a hypodivergent (*n* = 24) and a hyperdivergent (*n* = 18) group. All patients received a single-step mandibular advancement protocol through an FFA. Lateral cephalograms were analyzed after initial leveling and alignment (T1) and immediately after FFA removal (T2). The therapeutic effect was calculated through comparison with age-matched controls from a growth survey. Statistical significance was set at *p* < 0.05.

**Results:**

Hypodivergent and hyperdivergent patients showed different treatment outcomes, but significant differences existed only for overbite and interincisal angle. Nearly all measurements suggested similar treatment-related changes for both groups with exception for dentoalveolar parameters.

**Conclusion:**

Treatment with FFA causes similar skeletal and dentoalveolar effects in hypodivergent and in hyperdivergent patients. The correction of overjet and molar relationship is mainly caused by dentoalveolar changes.

**Clinical relevance:**

Hyperdivergent patients do not respond unfavorably to FFA treatment compared to hypodivergent patients. Lower incisor protrusion occurs more pronounced in hypodivergent patients. The growth pattern ought to be considered when choosing FFA for class II treatment.

## Introduction

Among other characteristics, a class II malocclusion has a distobasal jaw relation between mandible and maxilla. It is the most frequent skeletal sagittal disharmony [1] in Caucasian populations. Fixed functional appliances (FFA) are proven for class II treatment. Other than removable functional appliances (RFA), FFA act without patient compliance and ensure force application 24 h per day [2–4].

Several FFA types have been described in the literature [5]. Herbst and FMA are two widely acclaimed and frequently used FFA types. The most common FFA became the Herbst appliance [6], introduced by Emil Herbst in 1909. He published a series of three articles [7–9] in 1934, describing his experiences with this appliance. After that, the appliance left orthodontics for decades [3] until Pancherz rediscovered the Herbst appliance and continued related research [10] from 1979 on. The Herbst appliance consists of a telescopic (rod and tube) mechanism which rigidly connects the first maxillary molars with the mandibular first bicuspids in order to exert continuous advancing forces on the mandible [3, 11].

The Functional Mandibular Advancer (FMA) was introduced by Kinzinger et al. [12] in 2002. The FMA exerts a rigid intergnathic force between upper and lower first molars for mandibular advancement. It comprises inclined planes at 60° to horizontal, thus adopting an established concept from functional orthodontics [11, 13]. Cast splint variants of Herbst appliance and FMA are used if improved dental anchorage is desired [6, 12].

Treatment effects have been documented in several investigations for both Herbst appliance [14, 15] and FMA [16, 17] and were found to be quite similar [11, 18, 19]. However, patients with hypodivergent or hyperdivergent facial growth patterns may respond differently to FFA treatment since hyperdivergent patients may respond unfavorably to FFA treatment [20]. Hyperdivergent facial growth thus appears as relative contraindication for FFA treatment. However, studies on FFA-treatment effects in hypodivergent or hyperdivergent growing patients are only scarcely available for the Herbst appliance [4, 21] and non-existent for the FMA.

The aims of this study were to investigate and compare the effects of orthodontic treatment with cast splint fixed functional appliance (FFA) in patients with hypo and hyperdivergent growth patterns, including possible side effects on occlusal plane, gonial angle, and lower incisor inclination.

## Material and methods

### Patients

*N* = 47 patients received treatment by the same experienced orthodontist with a cast splint fixed functional appliance (FFA) because of a skeletal class II malocclusion.

The inclusion criteria were as follows:

- Full permanent dentition (except for third molars),
- No tooth loss during treatment,
- No previous orthodontic treatment,
- Pre-treatment ANB ≥ 4° and distal occlusion of at least ½ cusp width,
- Caucasian origin of patients (visual inspection).

The exclusion criteria were as follows:

- Craniofacial anomalies,
- Loss or agenesis of permanent teeth (except for third molars),
- Extraction therapy.

No age restrictions were applied. *N* = 47 patients were screened for eligibility. *N* = 42 patients were included after 5 patients dropped out due to various reasons. The examination was performed on each patient at T1 and T2, with T1 recorded after initial leveling and alignment before and T2 immediately after FFA removal. The patients were divided into a hypodivergent group (*n* = 24, 11 m, 13 f, age at T1 14.76 ± 4.29 years) and a hyperdivergent group (*n* = 18, 11 m, 7 f, age at T1 14.10 ± 4.19 years). No significant age difference existed (*p* = 0.622). The division into a hypodivergent or hyperdivergent group followed a study by Rogers et al. [4] and depended upon the pre-treatment mandibular plane angle < 34° or ≥ 34°. At T1, the mandibular plane angle was significantly (*p* < 0.001) different between the groups.

The sample size was calculated in agreement with a similar study [22], based on a significance level of 0.05 and a power of 80% to detect a clinically meaningful difference of 2.0 (± 2.0 mm/degrees). The power analysis revealed that 17 patients were necessary for each group.

A control group was created from data of the growth study by Bhatia and Leighton [23] to ensure comparability to other Herbst and FMA studies [24, 25]. The data from Caucasian subjects were collected in a longitudinal survey of facial growth at King’s College in London/UK [25]. The chronological patient age was recorded instead of the of skeletal maturation stage in an earlier investigation [17]. The corresponding values for the control group were then matched to patient age at T1 and T2. The difference between T1 and T2 in the control group represented natural growth effects, unaffected by orthodontic treatment. This difference was subtracted from the delta between T1 and T2 in the study groups. The resulting values then represented the treatment effect, referred to as *Net* effect.

All patients received a fixed functional appliance based on their choice after seeing pictures of the appliances, either cast splint FMA (Functional Mandibular Advancer®, Forestadent, Pforzheim, Germany) or cast splint Herbst appliance [6] (Herbst®, Dentaurum, Ispringen, Germany). Since treatment effects of Herbst appliance and FMA proved to be rather similar [11, 18, 19], Herbst and FMA patients were pooled for the present study. A single-step-protocol was used for mandibular advancement into an edge-to-edge position. Treatment was exclusively performed by one experienced orthodontist.

### FFA treatment protocol

All patients received comprehensive fixed appliance treatment with an “MBT 0.022” bracket system. Dental arches were leveled and aligned up to a “0.017 × 0.025” stainless steel wire. After partial removal of the fixed appliance from the posterior dentition, the cast splint FFA was inserted. After successful FFA treatment, the FFA was removed again, and brackets were reattached to the posterior dentition in order to complete finishing and retention.

### Lateral cephalograms

Lateral cephalograms were available for all patients after initial leveling and alignment (T1) and immediately after FFA removal (T2). All cephalograms were recorded with an analog X-ray machine (Orthophos®, Sirona, Bensheim, Germany) with standardized conditions regarding head posture and maximal intercuspation. All images included a scale for the calculation of the enlargement factor. The radiation data varied between 73 kV/15 mA and 77 kV/14 mA depending on patient height and weight, and exposure time was always 9 s. The lateral cephalograms were digitized and analyzed using dedicated tracing software with an accuracy of two decimals on a certified image viewing system for radiographic diagnostics.

**Fig. 1:**
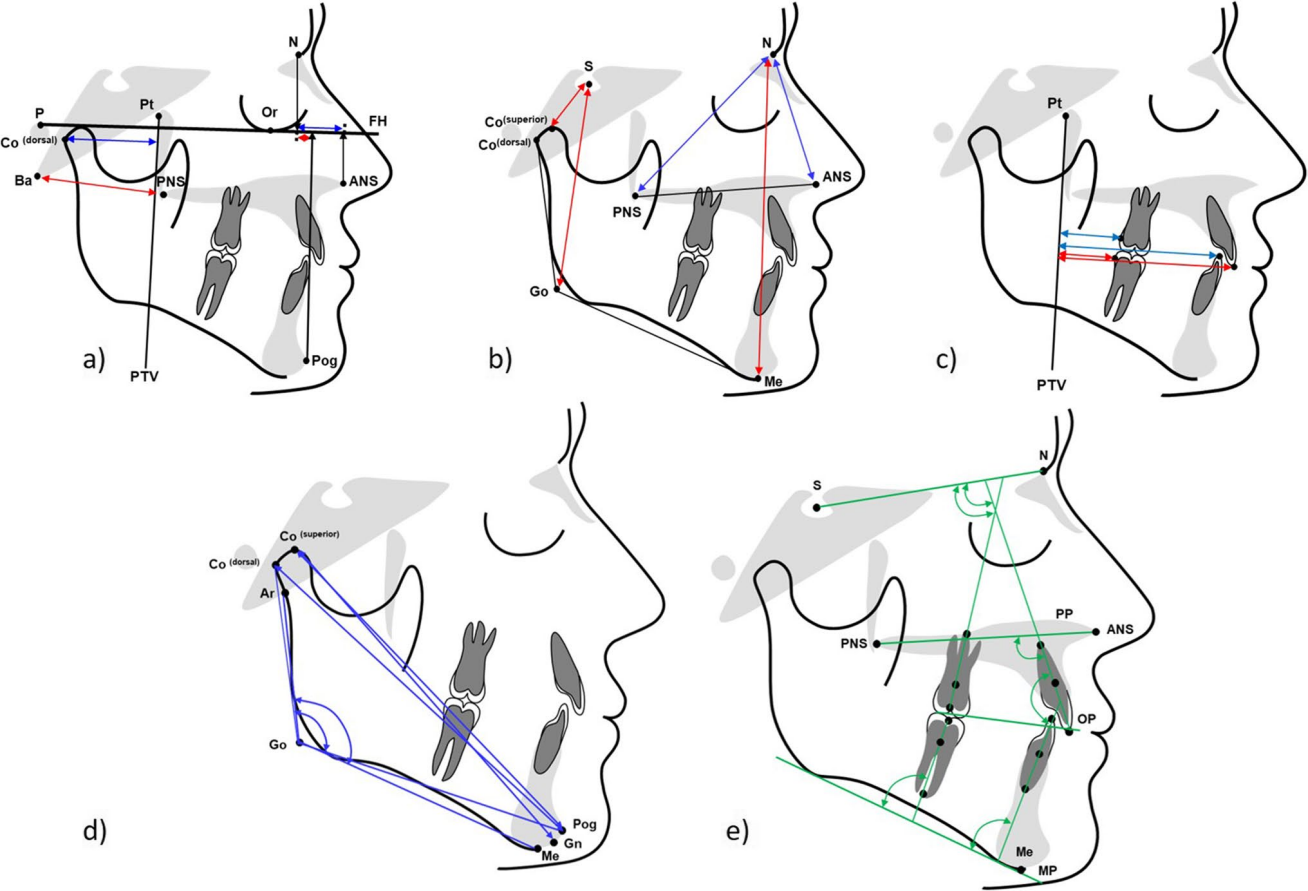
Skeletal and dental cephalometric measurements. **a** Horizontal linear: Co^(dorsal)−^PTV; Ba-PNS; N-ANS on FH; N-Pog on FH. **b** Vertical linear: S-Co^(superior)^; S-Go; N-Me; N-ANS; N-PNS. **c** Dentoalveolar linear: U1^(incisal)^-PTV; L1^(incisal)^-PTV; U6^(dorsal)^-PTV; L6^(dorsal)^-PTV. **d** Mandibular angular and linear: Co^(dorsal)^-Pog; Co^(superior)^-Gn; Ar-Go-Me; Co^(dorsal)^-Go-Pog (»modified gonial angle«). **e** Dentoalveolar angular: SN/OP; U1/SN; U1/PP; L1/MP; U1/L1 (interincisal angle); U6/SN; L6/MP

Hand-wrist X-rays were not taken, respecting the ALARA [26] principle. The control group data [23] also used chronological age rather than stages of skeletal maturity. The lateral cephalograms were analyzed according to Kinzinger et al. [25, 27] by a single blinded examiner to ensure comparability with other studies. A dedicated tracing software was used (fr-win®, version 7.0, Computer Konkret, Falkenstein, Germany). Measurements are shown in Table 1 and Fig. 1. The analyses included maxillary and mandibular sagittal and vertical as well as sagittal dental parameters.

**Table 1:** Cephalometric landmarks and measurements

Measurement	I. Skeletal and dental effects
Maxilla sagittal (mm)	
N-ANS on FH	Anterior position of the maxillary base: linear distance between the junction of the frontal bone and nasal bone at the nasofrontal suture (Nasion (N)) and the most anterior point of the bony floor of the nose at the tip of the anterior nasal spine (ANS) projected onto the Frankfurt Horizontal (FH)
Ba-PNS	Posterior position of the maxillary base: linear distance between the anterior margin of the foramen magnum (Basion (Ba)) and most posterior point of the bony floor of the nose at the tip of the posterior nasal spine (PNS)
Maxilla vertical (mm)	
N-ANS	Linear distance between landmarks Nasion (N) and anterior nasal spine (ANS)
N-PNS	Linear distance between landmarks Nasion (N) and posterior nasal spine (PNS)
Mandible sagittal (mm)	
N-Pog on FH	Anterior position of the mandibular base: linear distance between landmark Nasion (N) and most anterior point of the bony chin (Pog) projected onto the Frankfurt Horizontal (FH)
Co^(dorsal)−^PTV	Position of the dorsal condyle margin: linear distance between the most posterior point of the mandibular condyle (Co^(dorsal)^) and pterygoid vertical (PTV)
Mandible vertical (mm)	
S-Co^(superior)^	Linear distance between the Sella turcica’s midpoint (Sella, (S)) and condyle’s superior margin (Co^(superior)^)
S-Go	Linear distance between landmark Sella (S) and intersection of the ramus tangent and corpus tangent (Go)
N-Me	Linear distance between landmark Nasion (N) and most inferior point of the bony chin (Me)
Dental horizontal (mm)	
U1^(incisal)^-PTV	Linear distance between the incisal tip of the upper central incisor (U1^(incisal)^) and PTV†
L1^(incisal)^-PTV	Linear distance between the incisal tip of the lower central incisor (L1^(incisal)^) and PTV†
U6^(dorsal)^-PTV	Linear distance between the most distal point of upper first molar’s tooth crown (U6^(dorsal)^) and PTV†
L6^(dorsal)^-PTV	Linear distance between the most distal point of lower first molar’s tooth crown (L6^(dorsal)^) and PTV†
Overjet	Distance between the incisal tips of the lower (L1^(incisal)^) and upper central incisors (U1^(incisal)^) measured along the occlusal plane (OP)
Overbite	Distance between the tips of the lower (L1^(incisal)^) and upper central incisors (U1^(incisal)^) measured perpendicular to the occlusal plane (OP)
	II. Side effects on occlusal plane, gonial angle, and lower incisor inclination
Mandible diagonal (mm)	
Co^(dorsal)^-Pog	Linear distance between landmarks Co^(dorsal)^ and Pog
Co^(superior)^-Gn	Linear distance between the most superior point of the mandibular condyle (Co^(superior)^) and most anterior, inferior point on the mandibular symphysis (Gnathion, (Gn))
Mandible angular (°)	
Ar-Go-Me	Gonial angle: angle between intersection of the posterior border of the neck of the condyle with the cranial base (Ar) and landmarks gonion (Go), and menton (Me)
Co^(dorsal)^-Go-Pog	Modified gonial angle: angle between the landmarks posterior condylar margin (Co^(dorsal)^), gonion (Go), and pogonion (Pog) landmarks
Cant of occlusal plane (°)	
SN/OP	Angle between the anterior cranial base (SN) and the occlusal plane (OP)
Dental angular (°)	
U1/SN	Angle between the longitudinal axis of the upper central incisor (U1) and anterior cranial base (SN)
U1/PP	Angle between the longitudinal axis of the upper central incisor (U1) and palatal plane (PP)
L1/MP	Angle between the longitudinal axis of the lower central incisor (L1) and mandibular plane (MP)
U1/L1	Interincisal angle: angle formed by the intersection of the longitudinal axis of the upper central incisor (U1) with the longitudinal axis of the lower central incisor (L1)
U6/SN	Angle between the longitudinal axis of the upper first molar (U6) and anterior cranial base (SN)
L6/MP	Angle between the longitudinal axis of the lower first molar (L6) and mandibular plane (MP)

^†^Measurement perpendicularly onto PTV

### Statistical analysis

Normal distribution of the data was confirmed by the Shapiro–Wilk test. Homogeneity of variance was tested using Levene’s method. One-sample Student’s *t*-tests were applied for intragroup comparisons, and independent Student’s *t*-tests for intergroup comparisons. Descriptive statistics mean (M) and standard deviation (SD) are recorded for each variable. Additionally, 95% confidence intervals (95% CI) were calculated. All statistical analyses were performed using SPSS® version 28 for Windows® (IBM Corp., Armonk, NY, USA). Statistical significance was set at *p* < 0.05.

A total of 25% of the lateral cephalograms were randomly selected and re-traced after one month by the same examiner. The method error (ME) was calculated using the Dahlberg-formula (ME = √(∑*d*^2^/2*n*)) [28]. ME was < 1 for linear (0.78 mm) and angular (0.57°) measurements.

## Results

FFA-treatment time was 1.77 ± 0.95 years in hypodivergent patients and 1.97 ± 0.96 years in hyperdivergent patients. No significant difference was found (*p* = 0.493).

All cephalometric measurements are shown for dentoalveolar (Tables 2 and 4) and skeletal (Tables 3 and 5) measurements in Tables 2, 3, 4, and 5.

**Table 2:** Skeletal and dental effects in hypodivergent patients. Means (M) and standard deviations (SD) in cephalometric measurements at T1 and T2; *Net* net outcome/therapeutic effect, *CI* confidence interval, *LB* lower bound, *UB* upper bound, *NS* not significant. *p < 0.05; **p < 0.01; ***p < 0.001, calculation of ΔT2–T1: positive value = increase; negative value = decrease

Fixed functional appliances—hypodivergent patients							
Measurement	T1 (M ± SD) 95% CI (LB, UB)	T2 (M ± SD) 95% CI (LB, UB)	∆T2 − T1 (M ± SD) 95% CI (LB, UB)	Control (M ± SD) 95% CI (LB, UB)	Net (M ± SD) 95% CI (LB, UB)	Net *p*-value (intra)	Net *p*-value (inter)
Maxilla sagittal (mm)							
N-ANS on FH	4.64 ± 1.93 3.83, 5.45	4.81 ± 2.97 3.56, 6.07	0.17 ± 2.36 -0.82, 1.17	0.26 ± 0.48 0.06, 0.46	−0.09 ± 2.38 −1.09, 0.92	0.860 ^NS^	0.877 ^NS^
Ba-PNS	45.18 ± 2.83 43.98, 46.37	46.00 ± 2.71 44.86, 47.14	0.82 ± 2.23 −0.12, 1.77	0.57 ± 0.70 0.28, 0.87	0.25 ± 2.25 −0.70, 1.20	0.586 ^NS^	0.803 ^NS^
Maxilla vertical (mm)							
N-ANS	49.63 ± 4.34 47.80, 51.47	51.73 ± 4.05 50.02, 53.44	2.10 ± 2.88 0.88, 3.31	1.09 ± 1.13 0.61, 1.57	1.01 ± 2.46 −0.03, 2.05	0.057 ^NS^	0.675 ^NS^
N-PNS	69.16 ± 4.56 67.23, 71.09	71.28 ± 4.53 69.36, 73.19	2.12 ± 3.25 0.74, 3.49	1.18 ± 1.48 0.56, 1.81	0.93 ± 3.07 −0.36, 2.23	0.150 ^NS^	0.667 ^NS^
Mandible sagittal (mm)							
N-Pog on FH	−6.71 ± 5.43 −9.00, −4.42	−5.47 ± 6.31 −8.13, −2.80	1.24 ± 4.79 −0.78, 3.27	1.10 ± 1.16 0.61, 1.59	0.15 ± 4.73 −1.85, 2.14	0.881 ^NS^	0.696 ^NS^
Co^(dorsal)−^PTV	34.90 ± 2.45 33.86, 35.93	34.87 ± 3.10 33.56, 36.18	−0.03 ± 2.61 −1.13, 1.08	0.51 ± 0.59 0.26, 0.75	−0.53 ± 2.67 −1.66, 0.60	0.340 ^NS^	0.229 ^NS^
Mandible vertical (mm)							
S-Co^(superior)^	20.43 ± 2.30 19.46, 21.40	21.75 ± 3.10 20.44, 23.06	1.32 ± 2.45 0.29, 2.36	0.51 ± 0.61 0.25, 0.77	0.81 ± 2.45 −0.22, 1.84	0.119 ^NS^	0.319 ^NS^
S-Go	74.30 ± 6.82 71.42, 77.18	78.66 ± 6.91 75.74, 81.57	4.35 ± 3.06 3.06, 5.64	2.11 ± 2.19 1.18, 3.04	2.24 ± 2.82 1.06, 3.43	< 0.001 ^***^	0.222 ^NS^
N-Me	109.03 ± 7.16 106.01, 112.05	113.43 ± 6.97 110.48, 116.37	4.40 ± 4.74 2.39, 6.40	2.52 ± 2.38 1.52, 3.53	1.87 ± 4.22 0.09, 3.65	0.040 ^*^	0.389 ^NS^
Dental horizontal (mm)							
U1^(incisal)^-PTV	55.83 ± 5.29 53.59, 58.06	56.01 ± 3.97 54.34, 57.68	0.18 ± 3.82 −1.43, 1.79	1.25 ± 1.94 0.43, 2.07	−1.07 ± 3.94 −2.73, 0.60	0.197 ^NS^	0.464 ^NS^
L1^(incisal)^-PTV	48.65 ± 4.48 46.76, 50.54	52.93 ± 4.44 51.06, 54.81	4.28 ± 3.08 2.98, 5.58	1.24 ± 1.92 0.43, 2.05	3.04 ± 2.92 1.81, 4.28	< 0.001 ^***^	0.649 ^NS^
U6^(dorsal)^-PTV	15.56 ± 4.75 13.56, 17.57	15.94 ± 3.55 14.44, 17.44	0.38 ± 2.79 −0.80, 1.56	1.25 ± 1.94 0.43, 2.07	−0.87 ± 3.12 −2.19, 0.44	0.184 ^NS^	0.597 ^NS^
L6^(dorsal)^-PTV	13.39 ± 4.62 11.44, 15.34	16.46 ± 4.08 14.74, 18.19	3.07 ± 3.60 1.55, 4.59	1.27 ± 1.98 0.44, 2.11	1.80 ± 3.47 0.33, 3.27	0.018 ^*^	0.881 ^NS^
Overjet	6.94 ± 2.72 5.80, 8.09	2.52 ± 1.20 2.02, 3.03	−4.42 ± 2.40 −5.44, −3.41	−0.11 ± 0.19 −0.20, −0.03	−4.31 ± 2.39 −5.32, −3.30	< 0.001 ^***^	0.448 ^NS^
Overbite	3.68 ± 1.39 3.09, 4.26	1.97 ± 1.07 1.52, 2.42	−1.70 ± 1.14 −2.18, −1.22	0.03 ± 0.26 −0.08, 0.14	−1.73 ± 1.11 −2.20, −1.26	< 0.001 ^***^	0.004 ^**^

**Table 3:** Side effects on occlusal plane, gonial angle, and lower incisor inclination in hypodivergent patients. Means (M) and standard deviations (SD) in cephalometric measurements at T1 and T2; *Net* net outcome = therapeutic effect, *CI* confidence interval, *LB* lower bound, *UB* upper bound, *NS* not significant. *p < 0.05; **p < 0.01; ***p < 0.001, calculation of ΔT2–T1: positive value = increase; negative value = decrease

Fixed functional appliances—hypodivergent patients							
Measurement	T1 (M ± SD) 95% CI (LB, UB)	T2 (M ± SD) 95% CI (LB, UB)	∆T2 − T1 (M ± SD) 95% CI (LB, UB)	Control (M ± SD) 95% CI (LB, UB)	Net (M ± SD) 95% CI (LB, UB)	Net *p*-value (intra)	Net *p*-value (inter)
Mandible diagonal (mm)							
Co^(dorsal)^-Pog	104.39 ± 7.47 101.23, 107.55	107.84 ± 7.41 104.71, 110.97	3.45 ± 3.66 1.90, 5.00	1.34 ± 1.32 0.79, 1.90	2.11 ± 3.94 0.44, 3.77	0.015 ^*^	0.683 ^NS^
Co^(superior)^-Gn	107.05 ± 7.89 103.72, 110.38	111.22 ± 7.58 108.02, 114.42	4.17 ± 3.97 2.50, 5.85	3.10 ± 3.01 1.83, 4.37	1.08 ± 3.21 −0.28, 2.43	0.115 ^NS^	0.708 ^NS^
Mandible angular (°)							
Ar-Go-Me	120.91 ± 7.89 117.58, 124.24	120.80 ± 7.56 117.60, 123.99	−0.12 ± 4.11 −1.85, 1.62	−0.54 ± 0.40 −0.71, −0.37	0.42 ± 4.01 −1.27, 2.12	0.609 ^NS^	0.455 ^NS^
Co^(dorsal)^-Go-Pog	115.32 ± 6.88 112.42, 118.22	115.25 ± 7.43 112.11, 118.38	−0.07 ± 3.45 −1.53, 1.38	−0.54 ± 0.40 −0.71, −0.37	0.47 ± 3.39 −0.97, 1.90	0.507 ^NS^	0.524 ^NS^
Cant of occlusal plane (°)							
SN/OP	17.97 ± 3.81 16.36, 19.58	17.32 ± 3.69 15.76, 18.88	−0.65 ± 2.76 −1.81, 0.51	−0.99 ± 1.56 −1.65, −0.33	0.34 ± 2.91 −0.89, 1.57	0.577 ^NS^	0.156 ^NS^
Dental angular (°)							
U1 / SN	104.65 ± 7.70 101.40, 107.91	103.04 ± 6.38 100.35, 105.74	−1.61 ± 8.23 −5.09, 1.86	−0.04 ± 0.82 −0.38, 0.31	−1.58 ± 8.05 −4.98, 1.83	0.348 ^NS^	0.332 ^NS^
U1 / PP	113.53 ± 8.06 110.13, 116.93	111.11 ± 6.22 108.48, 113.74	−2.42 ± 7.31 −5.51, 0.67	0.02 ± 0.74 −0.29, 0.34	−2.44 ± 7.06 −5.42, 0.54	0.103 ^NS^	0.339 ^NS^
L1 / MP	101.49 ± 6.33 98.82, 104.17	107.66 ± 8.32 104.14, 111.17	6.17 ± 6.18 3.56, 8.78	−0.19 ± 0.41 −0.36, −0.01	6.35 ± 6.20 3.74, 8.97	< 0.001 ^***^	0.062 ^NS^
U1/ L1 (interincisal angle)	124.60 ± 11.87 119.58, 129.61	121.08 ± 9.32 117.14, 125.01	−3.52 ± 7.57 −6.72, −0.33	0.80 ± 1.04 0.36, 1.24	−4.32 ± 7.58 −7.52, −1.12	0.010 ^*^	0.031 ^*^
U6 / SN	69.47 ± 6.75 66.62, 72.32	70.81 ± 6.85 67.92, 73.71	1.34 ± 4.75 −0.66, 3.35	−0.05 ± 0.82 −0.40, 0.30	1.39 ± 4.75 −0.62, 3.40	0.348 ^NS^	0.076 ^NS^
L6 / MP	92.72 ± 3.60 91.20, 94.24	91.71 ± 6.35 89.03, 94.40	−1.01 ± 6.57 −3.78, 1.76	−0.16 ± 0.43 −0.34, 0.02	−0.85 ± 6.61 −3.64, 1.94	0.536 ^NS^	0.781 ^NS^

**Table 4:** Skeletal and dental effects in hyperdivergent patients. Means (M) and standard deviations (SD) in cephalometric measurements at T1 and T2; *Net* net outcome = therapeutic effect, *CI* confidence interval, *LB* lower bound, *UB* upper bound, *NS* not significant. *p < 0.05; **p < 0.01; ***p < 0.001, calculation of ΔT2–T1: positive value = increase; negative value = decrease

Fixed functional appliances—hyperdivergent patients							
Measurement	T1 (M ± SD) 95% CI (LB, UB)	T2 (M ± SD) 95% CI (LB, UB)	∆T2 − T1 (M ± SD) 95% CI (LB, UB)	Control (M ± SD) 95% CI (LB, UB)	Net (M ± SD) 95% CI (LB, UB)	Net *p*-value (intra)	Net *p*-value (inter)
Maxilla sagittal (mm)							
N-ANS on FH	4.11 ± 3.64 2.30, 5.92	4.08 ± 3.89 2.14, 6.01	−0.03 ± 3.31 −1.68, 1.61	0.19 ± 0.39 0.00, 0.39	−0.23 ± 3.48 −1.96, 1.50	0.785 ^NS^	0.877 ^NS^
Ba-PNS	44.33 ± 3.97 42.35, 46.30	45.54 ± 3.70 43.70, 47.38	1.21 ± 2.97 −0.27, 2.68	0.76 ± 0.77 0.37, 1.14	0.45 ± 2.85 −0.97, 1.87	0.512 ^NS^	0.803 ^NS^
Maxilla vertical (mm)							
N-ANS	51.22 ± 4.41 49.03, 53.41	53.40 ± 3.57 51.63, 55.18	2.18 ± 2.97 0.70, 3.66	1.48 ± 1.35 0.81, 2.15	0.70 ± 2.09 −0.34, 1.74	0.172 ^NS^	0.675 ^NS^
N-PNS	70.94 ± 6.28 67.82, 74.06	73.11 ± 4.78 70.73, 75.49	2.17 ± 3.46 0.45, 3.89	1.62 ± 1.58 0.83, 2.40	0.55 ± 2.46 −0.67, 1.77	0.354 ^NS^	0.667 ^NS^
Mandible sagittal (mm)							
N-Pog on FH	−7.82 ± 5.93 −10.77, −4.87	−7.43 ± 5.35 −10.09, −4.77	0.39 ± 4.15 −1.68, 2.45	0.83 ± 1.47 0.10, 1.57	−0.44 ± 4.91 −2.88, 2.00	0.706 ^NS^	0.696 ^NS^
Co^(dorsal)−^PTV	34.17 ± 3.03 32.66, 35.68	35.57 ± 4.98 33.09, 38.04	1.40 ± 4.68 −0.93, 3.72	0.60 ± 0.63 0.29, 0.91	0.80 ± 4.34 −1.36, 2.95	0.448 ^NS^	0.229 ^NS^
Mandible vertical (mm)							
S-Co^(superior)^	20.71 ± 4.44 18.50, 22.91	21.54 ± 3.99 19.55, 23.53	0.83 ± 2.15 −0.24, 1.90	0.73 ± 0.75 0.36, 1.10	0.10 ± 1.96 −0.87, 1.08	0.828 ^NS^	0.319 ^NS^
S-Go	70.95 ± 7.19 67.38, 74.53	74.99 ± 6.65 71.69, 78.30	4.04 ± 3.81 2.15, 5.94	2.78 ± 2.29 1.64, 3.91	1.27 ± 2.06 0.25, 2.29	0.018 ^*^	0.222 ^NS^
N-Me	116.43 ± 10.48 111.21, 121.64	120.54 ± 8.27 116.42, 124.65	4.11 ± 4.82 1.71, 6.51	3.32 ± 2.74 1.96, 4.68	0.79 ± 3.65 −1.02, 2.60	0.371 ^NS^	0.389 ^NS^
Dental horizontal (mm)							
U1^(incisal)^-PTV	55.67 ± 2.95 54.20, 57.13	55.38 ± 3.73 53.52, 57.23	−0.29 ± 2.57 −1.57, 0.99	1.60 ± 2.16 0.53, 2.67	−1.89 ± 2.98 −3.37, −0.41	0.015 ^*^	0.464 ^NS^
L1^(incisal)^-PTV	47.97 ± 2.77 46.59, 49.35	52.20 ± 3.73 50.35, 54.05	4.23 ± 2.72 2.88, 5.59	1.60 ± 2.16 0.53, 2.67	2.63 ± 2.82 1.23, 4.03	0.001 ^**^	0.649 ^NS^
U6^(dorsal)^-PTV	14.18 ± 3.67 12.36, 16.01	15.09 ± 3.12 13.54, 16.64	0.90 ± 2.40 −0.29, 2.10	2.38 ± 3.94 0.42, 4.33	−1.47 ± 4.17 −3.54, 0.60	0.152 ^NS^	0.597 ^NS^
L6^(dorsal)^-PTV	11.97 ± 4.14 9.91, 14.03	15.52 ± 3.37 13.84, 17.20	3.55 ± 2.91 2.10, 5.00	1.60 ± 2.16 0.53, 2.67	1.95 ± 2.80 0.56, 3.34	0.009 ^**^	0.881 ^NS^
Overjet	7.48 ± 1.68 6.65, 8.32	2.55 ± 1.14 1.98, 3.12	−4.93 ± 1.58 −5.72, −4.15	−0.13 ± 0.19 −0.23, −0.04	−4.80 ± 1.51 −5.55, −4.05	0.009 ^**^	0.448 ^NS^
Overbite	2.01 ± 1.90 1.06, 2.95	1.97 ± 1.43 1.26, 2.68	−0.04 ± 1.81 −0.94, 0.86	0.22 ± 0.39 0.02, 0.41	−0.26 ± 2.03 −1.27, 0.75	0.596 ^NS^	0.004 ^**^

**Table 5:** Side effects on occlusal plane, gonial angle, and lower incisor inclination in hyperdivergent patients. Means (M) and standard deviations (SD) in cephalometric measurements at T1 and T2; *Net* net outcome = therapeutic effect, *CI* confidence interval, *LB* lower bound, *UB* upper bound, *NS* not significant. *p < 0.05; **p < 0.01; ***p < 0.001, calculation of ΔT2–T1: positive value = increase; negative value = decrease

Fixed functional appliances—hyperdivergent patients							
Measurement	T1 (M ± SD) 95% CI (LB, UB)	T2 (M ± SD) 95% CI (LB, UB)	∆T2 − T1 (M ± SD) 95% CI (LB, UB)	Control (M ± SD) 95% CI (LB, UB)	Net (M ± SD) 95% CI (LB, UB)	Net *p*-value (intra)	Net *p*-value (inter)
Mandible diagonal (mm)							
Co^(dorsal)^-Pog	104.76 ± 8.03 100.76, 108.75	109.48 ± 6.16 106.41, 112.54	4.72 ± 4.95 2.26, 7.18	2.09 ± 2.36 0.91, 3.26	2.63 ± 4.33 0.48, 4.79	0.019 ^*^	0.683 ^NS^
Co^(superior)^-Gn	107.96 ± 8.71 103.63, 112.30	113.08 ± 6.87 109.67, 116.50	5.12 ± 4.93 2.67, 7.57	3.67 ± 3.05 2.15, 5.19	1.45 ± 3.10 −0.09, 2.99	0.064 ^NS^	0.708 ^NS^
Mandible angular (°)							
Ar-Go-Me	128.59 ± 3.88 126.66, 130.53	129.51 ± 3.73 127.65, 131.36	0.91 ± 4.12 −1.14, 2.96	−0.48 ± .31 −0.63, −0.32	1.39 ± 4.19 −0.70, 3.47	0.178 ^NS^	0.455 ^NS^
Co^(dorsal)^-Go-Pog	122.68 ± 4.51 120.43, 124.92	123.37 ± 3.75 121.50, 125.23	0.69 ± 3.52 −1.06, 2.44	−0.48 ± 0.31 −0.63, −0.32	1.16 ± 3.60 −0.63, 2.96	0.188 ^NS^	0.524 ^NS^
Cant of occlusal plane (°)							
SN/OP	19.79 ± 4.95 17.33, 22.25	20.31 ± 5.28 17.68, 22.93	0.52 ± 3.00 −0.98, 2.01	−1.10 ± 1.25 −1.72, −0.48	1.61 ± 2.71 0.26, 2.96	0.022 ^*^	0.156 ^NS^
Dental angular (°)							
U1/SN	104.93 ± 7.84 101.04, 108.83	101.17 ± 8.67 96.86, 105.48	−3.77 ± 6.23 −6.87, −0.67	0.04 ± 0.91 −0.41, 0.50	−3.81 ± 6.13 −6.86, −0.76	0.017 ^*^	0.332 ^NS^
U1/PP	113.90 ± 6.85 110.49, 117.31	109.68 ± 7.94 105.74, 113.63	−4.22 ± 5.98 −7.19, −1.24	0.20 ± 0.95 −0.27, 0.67	−4.42 ± 5.80 −7.31, −1.53	0.005 ^**^	0.339 ^NS^
L1/MP	96.20 ± 5.65 93.39, 99.01	99.53 ± 5.59 96.75, 102.31	3.33 ± 4.44 1.12, 5.54	0.35 ± 0.88 −0.09, 0.79	2.98 ± 4.75 0.62, 5.34	0.016 ^*^	0.062 ^NS^
U1/L1 (interincisal angle)	119.77 ± 7.41 116.08, 123.45	121.11 ± 7.88 117.19, 125.03	1.34 ± 7.37 −2.32, 5.01	0.38 ± 1.06 −0.15, 0.91	0.97 ± 7.62 −2.82, 4.75	0.597 ^NS^	0.031 ^*^
U6/SN	67.07 ± 4.67 64.74, 69.39	65.72 ± 6.75 62.36, 69.08	−1.34 ± 5.12 −3.89, 1.20	0.04 ± 0.91 −0.41, 0.50	−1.39 ± 5.06 −3.90, 1.13	0.261 ^NS^	0.076 ^NS^
L6/MP	97.48 ± 7.29 93.86, 101.11	97.52 ± 5.95 94.56, 100.48	0.03 ± 5.18 −2.54, 2.61	0.35 ± 0.88 −0.09, 0.79	−0.32 ± 5.31 −2.96, 2.33	0.804 ^NS^	0.781 ^NS^

### Skeletal and dentoalveolar effects

Although measurements in hypodivergent and hyperdivergent patients result in different mean values, only changes of overbite and interincisal angle were significant. In hypodivergent patients, posterior (S-Go) and anterior face height (N-Me) increased significantly, whereas overbite decreased significantly. In hyperdivergent patients, posterior (S-Go) and anterior face height (N-Me) also increased, showing significant differences only for posterior face height. The measurements revealed an almost parallel increase of anterior and posterior lower face height in hypodivergent patients, whereas hyperdivergent patients had a significant posterior and an insignificant anterior face height increase. Other than in hypodivergent patients, the overbite remained almost unchanged.

### Effects on occlusal plane, gonial angle, and lower incisor inclination

In hypodivergent patients, mandibular length (Co^(dorsal)^-Pog) increased significantly. The lower incisors always showed significant protrusion, which was more expressed than in hyperdivergent patients. Overjet was corrected in hypodivergent and in hyperdivergent patients by significant maxillary and mandibular changes, including forward movement of the lower dentition. In all patients, the interincisal angle decreased accordingly. The occlusal plane presented anterior canting in both groups. This was more pronounced in hyperdivergent patients, but still not significant.

## Discussion

Literature data on FFA-treatment effects distinguishing between hypodivergent and hyperdivergent patients are scarcely available [4, 21]. A direct comparison of existing data with the present results was impossible due to different methodology.

### Methods

The *Net* effect was calculated by including data of a longitudinal survey of unaffected facial growth [23]. These data were obtained from a non-homogeneous sample of Caucasian individuals. These individuals were between 4 and 20 years old, but only age-matched data were used for our study. The ideal control with untreated class II subjects followed up on a regular basis is and will be unavailable. Therefore, limitations have to be acknowledged when employing data from growth studies [65].

It was criticized [32] that in some studies, patients undergoing either FMA or Herbst appliance treatment were pooled instead of evaluated separately [17, 27], and none of those studies included clear information regarding appliance activations [17, 25, 27]. Differences in design and biomechanical concepts of FMA and Herbst appliance allow the assumption that treatment effects are different as well. However, treatment-related effects have been documented in different investigations for both Herbst appliance [14, 15] and FMA [16, 17] and were found to be rather similar [11, 18, 19]. Hence, it is unlikely that patient pooling has influenced the results of the present study.

### Results

The present results showed that FFA treatment lead to an increase of total posterior face height (S-Go) and total anterior face height (N-Me) in both hypodivergent and hyperdivergent patients. Vertical increases for the posterior (1.4–2.5 mm) and anterior facial heights (1.2–3 mm) have already been reported [29]. The present results were well within those ranges.

The linear distance between the center of the Sella turcica and the superior margin (S-Co^(superior)^) of the condyle exhibited only minor average changes below 1 mm in hypodivergent and hyperdivergent FFA patients, thus confirming findings of another study [27]. The results of measurements of the anterior and posterior position of maxillary base (N-ANS on FH, Ba-PNS) showed minor changes that are probably clinically insignificant. It may thus be assumed that neither a growth-inhibiting effect nor a treatment-related change in maxillary length occurs. Similar results were reported by Kinzinger and Diedrich [25], who found no adverse effects upon the maxilla or the maxillary base in patients treated with the FMA. Similar findings were also reported for the Herbst appliance [30].

In both hypodivergent and hyperdivergent patients, maxillary vertical parameters (N-ANS, N-PNS) always increase, although changes were insignificant. As previously reported [27], changes were very small with a maximum of 1.01 mm, and thus probably clinically negligible [25, 27].

The anterior limit of the mandibular base (N-Pog on FH) and the position of the dorsal condyle margin (Co^(dorsal)^-PTV) showed only minor insignificant changes. Those findings were also confirmed in a previous study [27]. It is a well-established fact that dentoalveolar compensation is the main contributor to class II correction [25] when using fixed functional appliances.

The findings of this study demonstrate this significant dentoalveolar contribution to overjet and molar correction which corresponds to information from the literature [31]. This has also been confirmed for FFA treatment [17, 25, 31]. Contrary to that, Aras et al. [32] found no distal movement of maxillary molars. However, these authors attached an additional palatal arch to stainless steel crowns on the upper molars. Still, Aras et al. [32] also described mesial movement of the lower molars, confirming present and other results [17, 25].

A systematic review and meta-analysis by Zymperdikas et al. [33] concluded that the desired skeletal effects of FFA in class II patients excluding the effects of normal growth were small and probably clinically irrelevant [33].

An unrestricted comparison of literature data with the present results remains impossible, since no other authors considered natural growth effects over a comparable time span and used different landmarks and reference planes.

In all patients, the mandibular length increased, but significantly only in the hyperdivergent group. Mandibular lengthening during treatment with FFA was confirmed in numerous studies [10, 34–39]. The increase of Co^(dorsal)^-Pog distance in the present study was less than 3 mm, thus smaller than previously reported values between 3.0 and 7.5 mm [10, 34, 39, 40]. The significant increase in Co^(dorsal)^-Pog distance in all our patients after advancement of the mandible into the desired position suggests that the treatment leads to remodeling of the mandibular bone and temporomandibular joints [21, 41, 42].

The Co^(superior)^-Gn distance showed an average increase of 1.08 mm in hypodivergent and 1.45 mm in hyperdivergent patients. This is less than other reported values, which ranged between 3.4 and 6.6 mm [35–38]. The difference between the present findings and other studies [10, 34–39] might be explained by variations of the pre-treatment severity of class II malocclusion in the investigated patients.

The hyperdivergent patients showed increases of the gonial (Ar-Go-Me) and»modified« gonial angle (Co^(dorsal)^-Go-Pog) by 1.39° and 1.16°. These values were larger than those in another FMA investigation with average values below 1° [25].

The present hypodivergent patients showed no changes of the gonial angle which was also reported in previous investigations [10, 43]. Other investigators observed an increase of the gonial angle between 2.0 and 5.0°, but reported a complete relapse during the posttreatment period [44, 45]. Other studies described a further decrease of gonial angles as long-term change after Herbst appliance treatment between 1.0 [46] and 7.7° [44, 47]. Although not statistically significant, the hyperdivergent patients exhibited a mean increase of the gonial angle greater than 1° while hypodivergent patients showed no change.

Finite element model (FEM) simulations [48] could help to explain the treatment-related changes of the gonial angle as well as the difference between intergnathic force vectors exerted by the FFA in hypodivergent and hyperdivergent patients. Stress, displacement, and deformation of the mandible under different loads have been evaluated with FEM simulations [49–53]. The elastic properties of the human mandible [54, 55] explained mandibular deformation in different spatial directions [49–52]. It appears reasonable that rigid FFA might contribute to an increase of the gonial angle through mandibular deformation during treatment [49]. Until today, only two FEM analyses [56, 57] studied treatment-related effects of FFA. Still, none of these investigated possible changes of the gonial angle during a simulated mandibular protraction. Although an FEM analysis helps in understanding therapeutic effects, it remains an in vitro study model with restrictions concerning the replication of clinical conditions. Hence, the results may only be acknowledged qualitatively [48].

The present studies investigated only cast splint FMA and Herbst appliance variants. An increased inhibition of mandibular spatial deformation occurs if more teeth are splinted and more rigid attachments are used [58, 59]. Different changes in gonial angle after FMA or Herbst appliance might thus be attributed to the number of splinted mandibular teeth (3 versus 4) and to different concepts of intergnathic force application (»molar to molar« versus»molar to first premolar«). However, it is unlikely that these factors have influenced the present results because other investigations pointed out similar therapeutic effects of FMA and Herbst appliance [11, 18, 19].

In hypodivergent patients, the gonial angle remained nearly unchanged which might be attributed to the rigid cast splint that counteracts mandibular deformation. This might explain differences to findings from other investigations which observed an increase of the gonial angle in Herbst appliance patients between 2.0 and 5.0° [44, 45]. These studies investigated treatment with a Herbst appliance variant attached to orthodontic bands only [44]. Compared to cast splint FFA, banded variants are less rigid [6] and thus might not counteract to mandibular deformation to the same extent. Weschler and Pancherz compared [60] three different mandibular anchorage forms in Herbst appliance treatment, but did not measure the gonial angle, thus leaving no data for comparison.

Geometrical reasons require assessment of mandibular lengthening related to changes of the gonial angle. Any increase leads to caudal and dorsal displacement of the cephalometric landmarks Pogonion (Pog) and Gnathion (Gn). Hence, treatment-related mandibular lengthening might be underrated in linear sagittal measurements and overrated in linear oblique measurements. The present results suggest that mandibular lengthening in hypodivergent patients was hardly affected by gonial angle increase. In contrast, the treatment-related increase in the gonial angle was on average three times greater in hyperdivergent patients than in hypodivergent patients. This suggests that results of linear cephalometric measurements of the mandible were particularly affected by the growth pattern.

Pronounced dentoalveolar changes were expected and have occurred. Both hypodivergent and hyperdivergent patients showed retrusion of upper incisors and significant protrusion of lower incisors. Remarkably, hypodivergent patients showed more than twice as much (6.35° versus 2.98°) protrusion than hyperdivergent patients, contributing substantially to the decrease of the interincisal angle. These figures are very similar to those of other investigations. The greater lower incisor protrusion in hypodivergent patients may be attributed to a more horizontal force vector exerted by the rigid FFA. The mesially directed force of fixed functional appliances upon the mandibular dentition always causes lower incisor proclination. If periodontal problems are present, this might limit the indications for this therapy [27].

To counteract protrusion of lower incisors, the use of orthodontic miniplates or orthodontic miniimplants (OMI) has been described [22, 61–63]. Despite connecting a Herbst appliance to OMI by different types of ligation, the protrusion of lower incisors could not entirely be prevented [64]. Other authors [22, 61–63] connected their FFA to orthodontic miniplates attached to the bony chin. They were successful in class II correction due to pronounced skeletal effects without proclination of lower incisors. On the contrary, even retrusion of lower incisors was observed [22, 61]. It was deemed possible that the pressure of the upper incisors and lower lip caused this change [22]. However, besides possible advantages of this approach, the additional surgical procedures are disadvantageous for the patient [61].

The present results showed antero-caudal canting of the occlusal plane in all groups. However, this occurred significantly greater in hyperdivergent than in hypodivergent patients. Still, intergroup comparison between hypodivergent and hyperdivergent patients did not reveal significant differences. When including growth effects, a canting of the occlusal plane in relation to the sella-nasion-line increased only slightly by 0.34 mm in hypodivergent patients and by 1.61 mm in hyperdivergent patients. Kinzinger et al. [11] investigated dental and skeletal effects in FMA and Herbst patients, not only differentiating between hypodivergent and hyperdivergent patients but also considering growth effects. In their investigation, occlusal plane canting showed insignificant increases of 0.64 in FMA patients and 1.13 in Herbst patients, which was less than in the present study. However, the difference was only small and is probably clinically negligible.

## Conclusions

- Dentoalveolar rather than skeletal changes are responsible for improvements of overjet and occlusion in FFA treatment in hypodivergent and hyperdivergent patients.
- Lower incisor protrusion is inevitable and happened more pronounced in hypodivergent patients.
- Lower incisor protrusion may be a contraindication for FFA treatment, especially in hypodivergent patients.
- Effects of FFA treatment upon occlusal plane and gonial angle are negligible.
- A hyperdivergent growth pattern is no contraindication for FFA treatment.

## Informed consent

For this type of study, formal consent is not required.

## Competing interests

The authors declare no competing interest.

## Data Availability

Not applicable.
